# *Pueraria lobata* and Daidzein Reduce Cytotoxicity by Enhancing Ubiquitin-Proteasome System Function in SCA3-iPSC-Derived Neurons

**DOI:** 10.1155/2019/8130481

**Published:** 2019-10-07

**Authors:** I-Cheng Chen, Kuo-Hsuan Chang, Yi-Jing Chen, Yi-Chun Chen, Guey-Jen Lee-Chen, Chiung-Mei Chen

**Affiliations:** ^1^Department of Neurology, Chang Gung Memorial Hospital, Chang Gung University College of Medicine, Taoyuan 33302, Taiwan; ^2^Department of Life Science, National Taiwan Normal University, Taipei 11677, Taiwan

## Abstract

Spinocerebellar ataxia type 3 (SCA3) is an autosomal dominant neurodegenerative disorder caused by a CAG repeat expansion within the *ATXN3*/*MJD1* gene. The expanded CAG repeats encode a polyglutamine (polyQ) tract at the C-terminus of the ATXN3 protein. ATXN3 containing expanded polyQ forms aggregates, leading to subsequent cellular dysfunctions including an impaired ubiquitin-proteasome system (UPS). To investigate the pathogenesis of SCA3 and develop potential therapeutic strategies, we established induced pluripotent stem cell (iPSC) lines from SCA3 patients (SCA3-iPSC). Neurons derived from SCA3-iPSCs formed aggregates that are positive to the polyQ marker 1C2. Treatment with the proteasome inhibitor, MG132, on SCA3-iPSC-derived neurons downregulated proteasome activity, increased production of radical oxygen species (ROS), and upregulated the cleaved caspase 3 level and caspase 3 activity. This increased susceptibility to the proteasome inhibitor can be rescued by a Chinese herbal medicine (CHM) extract NH037 (from *Pueraria lobata*) and its constituent daidzein via upregulating proteasome activity and reducing protein ubiquitination, oxidative stress, cleaved caspase 3 level, and caspase 3 activity. Our results successfully recapitulate the key phenotypes of the neurons derived from SCA3 patients, as well as indicate the potential of NH037 and daidzein in the treatment for SCA3 patients.

## 1. Introduction

Spinocerebellar ataxia type 3 (SCA3, also known as Machado-Joseph disease (MJD)) is an autosomal dominant neurodegenerative disease and thought to be the most common subtype of SCA worldwide [1, 2]. SCA3 is caused by an expanded CAG repeats within the *ATXN3*/*MJD1* gene on chromosome 14q32.1 [3]. The most common isoform of *ATXN3* expressed in the brain contains 11 exons and is translated into approximately 42 kDa ATXN3 protein depending on the length of CAG repeats encoding a polyglutamine (polyQ) tract at the C-terminus of the ATXN3 protein [4]. ATXN3 protein is a deubiquitinating enzyme that plays an important role in the ubiquitin-proteasome system (UPS) that regulates protein degradation [5]. The Josephin domain together with the ubiquitin-interacting motifs deubiquitinates proteins to regenerate reusable ubiquitin [5, 6]. Besides the role in protein degradation, ATXN3 regulates transcription by interacting with numerous key regulators of transcription and also by directly binding to DNA through a leucine zipper motif [7].

The expanded polyQ tract (>51 repeats) in ATXN3 causes conformational changes, resulting in formation of protein aggregates and altered ATXN3 function. In the brains of SCA3 patients, mutant ATXN3 protein tends to accumulate in neuronal cell nuclei [8]. However, cytosolic aggregates are also found in mutant ATXN3-overexpressing COS-7 cells, suggesting a widespread aggregation of polyQ-expanded ATXN3 protein [9]. Aggregates formed by polyQ-expanded ATXN3 result in subsequent cellular dysfunctions including transcriptional dysregulation, mitochondrial function, and misfolded protein clearance, eventually leading to cell death and neurodegeneration [10].

Up to now, there is no effective treatment available for polyQ disorders including SCA3. Activation of autophagy and UPS could be potential therapeutic targets for SCA3. Overexpression of BECLIN-1 improved the clearance of mutant ATXN3 in rats [11]. Treatment with rapamycin to upregulate autophagy in SCA3 mice could decrease mutant ATXN3 aggregation [12]. Recently, cordycepin, a bioactive compound in the fungi *Cordyceps* and a derivative of the nucleoside adenosine, was found to activate autophagy through AMPK phosphorylation to reduce behavioral and neuropathological phenotypes in SCA3 mice [13]. Given that impairment of UPS plays an important role in pathogenesis of polyQ diseases including SCA3 [14], activation of UPS has also been implicated as a probable therapeutic target for SCA3 [15, 16]. Recently, lines of evidence have shown that traditional Chinese medicine is a potential strategy for the treatment of neurodegenerative diseases. For example, *Ginkgo biloba* extract EGb 761 has been reported to increase proteasome activity and enhance degradation of aggregated protein in a Huntington's disease (HD) cellular model [17]. Tianma (*Rhizoma gastrodiae*) modulates proteins related to the UPS in differentiated SH-SY5Y cells [18]. Previously, we also found that *Pueraria lobata* (NH037) and its active constituent daidzein significantly enhanced UPS function and proteasome activity, reduced ATXN3 polyQ aggregation, improved neurite outgrowth, and displayed antiapoptotic effects in ATXN3/Q_75_-GFP 293/SH-SY5Y cell models, whereas NH037 and daidzein did not activate autophagy in a HEK293 cell model [19]. Through promoting UPS to provide neuroprotection, NH037 and daidzein could be a potential candidate for developing a therapeutic strategy of SCA3.

Up to now, the available treatment to halt the neurodegeneration of SCA3 is still absent. The lack of live neurons from patients to investigate the molecular details of neurodegeneration may be a bottleneck for drug research in SCA3. Recent progress in the induced pluripotent stem cell (iPSC) technology offers a unique opportunity to acquire neurons carrying an identical genetic background of SCA3 patients to investigate the potential pathogenesis and therapeutic strategies for SCA3. In this study, we established iPSCs from SCA3 patients (SCA3-iPSCs), which were differentiated into neurons characterized by polyQ aggregates and impaired UPS. We further examined the neuroprotective potentials of NH037 and daidzein on the neurons derived from SCA3-iPSCs. Our findings provide a new disease-specific model for SCA3, which can also serve as a platform to identify new treatments for neurodegenerative diseases.

## 2. Materials and Methods

### 2.1. Derivation of Human Skin Fibroblasts

Briefly, human skin fibroblasts were obtained from two normal controls (NC-1: 63-year-old female; NC-2: 31-year-old male) and two SCA3 patients (SCA3-1: 47-year-old female, 66 repeats; SCA3-2: 50-year-old male, 68 repeats). Isolation and cultivation of human skin fibroblasts were performed as described previously [20].

### 2.2. Retroviral Reprogramming and Generation of Human iPSC Lines

Human skin fibroblasts were reprogrammed by four factor combinations including OCT4, SOX2, KLF4, and c-MYC, and mouse embryonic fibroblast (MEF) feeder cells from 13.5 to 14.5 dpc CD1 mouse embryos were prepared using a standard method as previously described [21]. The reprogrammed human iPSCs were maintained on mitomycin C-deactivated MEF feeder cells in Knockout Dulbecco's modified Eagle medium (DMEM, Invitrogen, Waltham, MA, USA) supplemented with 20% Knockout Serum Replacement (KSR, Invitrogen), 8 ng/ml bFGF (Peprotech, Rocky Hill, NJ, USA), 50 U/ml penicillin, 50 mg/ml streptomycin, 1 mM sodium pyruvate, 1x MEM nonessential amino acid, 2 mM L-glutamine, and 0.1 mM 2-mercaptoethanol.

### 2.3. SCA3 Genotyping

Genomic DNA from iPSCs and parental fibroblasts was extracted by a DNA extraction kit (Qiagen, Hilden, Germany) and amplified by PCR analysis with *ATXN3* specific primers (forward: CCAGTGACTACTTTGATTCG, reverse: CTTACCTAGATCACTCCCAA). Annealing temperature was at 54°C, and the length of PCR fragments ranges from 235 bp to 403 bp (corresponding to 12~68 CAG repeats).

### 2.4. Immunocytochemistry

Briefly, cells were fixed in 4% paraformaldehyde for 20 min, permeabilized with 0.05% Triton X-100 in PBS for 20 min, and then blocked in 1% BSA for at least 1 hour. Cells were then incubated with primary antibodies and corresponding secondary antibodies for further examination. For detecting polyQ aggregation, primary antibodies 1C2 (1 : 200, Millipore, Burlington, MA, USA) and P62 (1 : 200, Santa Cruz, Dallas, TX, USA) were used. Images were captured by a fluorescence microscope (DMI4000 B, Leica, Wetzlar, Germany) and processed by MetaMorph microscopy automation and image analysis software (Molecular Devices, Sunnyvale, CA, USA).

### 2.5. Characterization of Human iPSC Colonies

Alkaline phosphatase activity of the reprogrammed iPSC colonies was tested using an alkaline phosphatase detection kit (Millipore) according to the manufacturer's instructions. Immunocytochemistry was performed to confirm pluripotency marker expression (OCT4, 1 : 200, and TRA-1-81, 1 : 200, Abcam, Cambridge, UK). RT-PCR, karyotyping, and hematoxylin and eosin staining (HE staining) of teratoma tissues were performed as previously described [17].

### 2.6. Monolayer Neuronal Differentiation from iPSC Lines

iPSC cultures were disaggregated using Accutase (Millipore) and preplated on gelatin for 1 hour at 37°C to remove MEFs. The feeder-free iPSCs were plated on Matrigel (BD, Franklin Lakes, NJ, USA)-coated dishes in MEF-conditioned iPSC medium spiked with 8 ng/ml of bFGF. The initial differentiation medium conditions were N2B27 medium (1 : 1 mixture of DMEM/F12 supplemented with modified N2 and Neurobasal medium supplemented with B27, all purchased from Invitrogen) spiked with 10 *μ*M of the TGF*β* inhibitor SB431542 (Sigma-Aldrich, St. Louis, MO, USA). Neural progenitors were obtained and maintained with bFGF (10 ng/ml) and EGF (10 ng/ml, Peprotech) in poly-L-lysine-coated plates then characterized by immunostaining with neural stem cell (NSC) markers NESTIN (1 : 200, DSHB, Iowa City, IA, USA) and PAX6 (1 : 200, Covance, Princeton, NJ, USA). After supplementation with 20 ng/ml BDNF (Peprotech), 0.2 mM ascorbic acid (Sigma-Aldrich), 10 ng/ml GDNF (Peprotech), and 1 ng/ml TGF*β*3 (Peprotech) for two weeks, matured neurons were confirmed by TUJ1 (1 : 500, Covance).

### 2.7. Trypan Blue Exclusion Assay

For the cell viability assay, 20 *μ*l of 0.4% trypan blue (Invitrogen) was mixed with 20 *μ*l cell suspension from each sample and viable cells that excluded trypan blue were determined by light microscopy.

### 2.8. Caspase 3 Activity Measurement

Cells were lysed in 1x lysis buffer by repeated cycles of freezing and thawing. Caspase 3 activity was measured with the caspase 3 assay kit according to the manufacturer's instructions (Sigma-Aldrich).

### 2.9. Proteasome Activity Assay

Proteasome activity was determined using a proteasome activity assay kit (Abcam) with chymotrypsin-like (SUC-LLVY-AMC) substrates according to the manufacturer's protocol. Fluorescence was read using a 380/460 nm filter set in a fluorometer (FLx800 fluorescence microplate reader, BioTek, Winooski, VT, USA).

### 2.10. Filter Trap Assay

Cells were lysed in RIPA buffer, and protein samples were filtered through a cellulose acetate membrane (0.2 *μ*m pore size) by Bio-Dot® Microfiltration Apparatus (Bio-Rad, Hercules, CA, USA). The membrane was blocked in PBS containing 10% nonfat dried milk overnight, and the mouse 1C2 antibody (1 : 500, Millipore) was used to detect aggregation.

### 2.11. Cellular Radical Oxygen Species (ROS) Analysis

To measure oxidative stress induced by MG132, a fluorogenic reagent (CellROX™ Deep Green, Molecular Probes, Eugene, OR, USA) was added to cells at a final concentration of 5 *μ*M and incubated at 37°C for 30 min. Cells were washed with PBS and fixed in 4% formaldehyde. Images were captured by a fluorescence microscope (DMI4000 B, Leica) and processed by MetaMorph microscopy automation and image analysis software (Molecular Devices).

### 2.12. Lactate Dehydrogenase (LDH) Cell Toxicity Assay

Culture media were collected, and 100 *μ*l of supernatant from each sample was transferred to a 96-well plate to determine the release of LDH by the LDH cytotoxicity assay kit (Cayman, Ann Arbor, MI, USA). The absorbance was read at 490 nm with a microplate reader (Multiskan GO, Thermo Scientific, Waltham, MA, USA).

### 2.13. Western Blot Analysis

Cells were lysed in lysis buffer (1 mM EDTA, 1 mM PMSF, 5 *μ*g/ml aprotinin, 150 mM NaCl, 50 mM Tris-HCl pH 7.4, 5 *μ*g/ml leupeptin, 1% sodium deoxycholate, 1% Triton X-100, and 0.1% SDS). After sonication and sitting on ice for 20 min, the lysates were centrifuged at 14000 × g for 10 min at 4°C. Protein concentrations were determined using the Bio-Rad protein assay kit (Bio-Rad), with albumin as standards. Total proteins (20~30 *μ*g) were electrophoresed on 10~12% SDS-polyacrylamide gel and transferred onto a nitrocellulose membrane (Bio-Rad) by reverse electrophoresis. After being blocked by 10% milk, the membrane is stained with caspase 3 (1 : 1000; Cell Signaling, Danvers, MA, USA), ubiquitin (1 : 500; Dako, Santa Clara, CA, USA), and TUJ1 (1 : 5000; Covance) primary antibodies. The immune complexes are detected using horseradish peroxidase-conjugated goat anti-mouse (Jackson ImmunoResearch, West Grove, PA, USA) or goat anti-rabbit (Rockland, Pottstown, PA, USA) IgG antibody (1 : 10000 dilution) and Immobilon™ Western Chemiluminescent HRP substrate (Millipore).

### 2.14. Lipid Peroxidation Malondialdehyde (MDA) Assay

Oxidative stress was evaluated by the MDA assay kit (Abcam). Cells were prepared and examined according to the manufacturer's protocol. The fluorescence was read at 532 nm in a microplate reader (Multiskan GO, Thermo Scientific).

### 2.15. Herbal Extract and Compounds/Chemicals

Chinese herbal extract NH037 (1 mg/ml as final working concentration) was provided by Sun Ten Pharmaceutical Co. (New Taipei City, Taiwan), and the preparation was performed as previously described [22]. Daidzein (50 *μ*M) and MG132 (2 *μ*M) were purchased from Sigma-Aldrich.

### 2.16. Statistical Analysis

For each set of values, data are represented as the mean ± standard deviation (SD) of three independent experiments. Differences between groups were evaluated by two-tailed Student's *t*-test or ANOVA (one-way and two-way) with a *post hoc* LSD test where appropriate. A *p* value less than 0.05 was considered statistically significant.

## 3. Results

### 3.1. Generation and Neural Differentiation of iPSCs from SCA3 Patients

**Figure 1:**
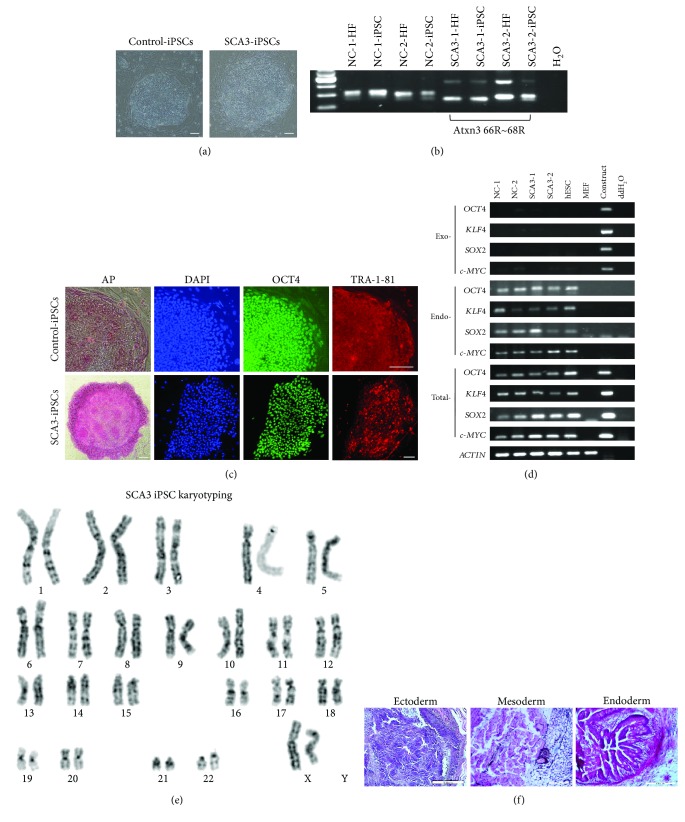
Characterization of SCA3 patient-specific-induced pluripotent stem cells (SCA3-iPSCs). (a) iPSC colonies obtained from reprogramming of normal controls and patients' fibroblasts. (b) Genotyping of iPSCs and fibroblasts (HF). PCR analysis showed SCA3 patients carrying expanded CAG repeats (66~68 repeats). (c) Immunofluorescent staining for the stem cell markers alkaline phosphatase (AP), OCT4 and TRA-1-81 in control- and SCA3-iPSCs. (d) RT-PCR analysis of exogenous and endogenous pluripotency markers (*OCT4*, *KLF4*, *SOX2*, and *c-MYC* genes) from iPSC cell lines. (e) Karyotyping analysis of SCA3-iPSCs. (f) Teratoma formed after subcutaneously injecting SCA3-iPSCs into nude mice. HE staining of teratomas showed endoderm, mesoderm, and ectoderm lineages. Scale bar: 100 *μ*m.

To establish iPSCs for SCA3, primary dermal fibroblasts from two SCA3 patients (SCA3-1: 47-year-old female; SCA3-2: 50-year-old male) and two normal controls (NC-1: 63-year-old female; NC-2: 31-year-old male) were transfected with four reprogramming factors OCT4, KLF4, SOX2, and c-MYC by retroviruses. Human ESC-like colonies were generated three to four weeks after transfection (Figure 1(a)). PCR analysis of genomic DNA showed that patients' iPSCs (SCA3-1 and SCA3-2) contain mutant *ATXN3* CAG repeats (SCA3-1 with 66 repeats and SCA3-2 with 68 repeats, Figure 1(b)). These iPSCs were positive for pluripotent markers alkaline phosphatase, OCT4, and TRA-1-81 (Figure 1(c)). RT-PCR analysis of iPSCs indicated that exogenous *OCT4*, *KLF4*, *SOX2*, and *c-MYC* genes delivered by retroviruses for reprogramming were silenced after passage. In contrast, endogenous pluripotent genes were highly expressed in NC- and SCA3-iPSC lines (Figure 1(d)). These iPSC lines demonstrated normal karyotypes (Figure 1(e)) and formed teratoma presenting with tissues belonging to three germ layers (Figure 1(f)). Selected iPSC clones from each line (NC-1, NC-2, SCA3-1, and SCA3-2) with good morphological features and absent expression of exogenous genes were used for further investigation.

**Figure 2:**
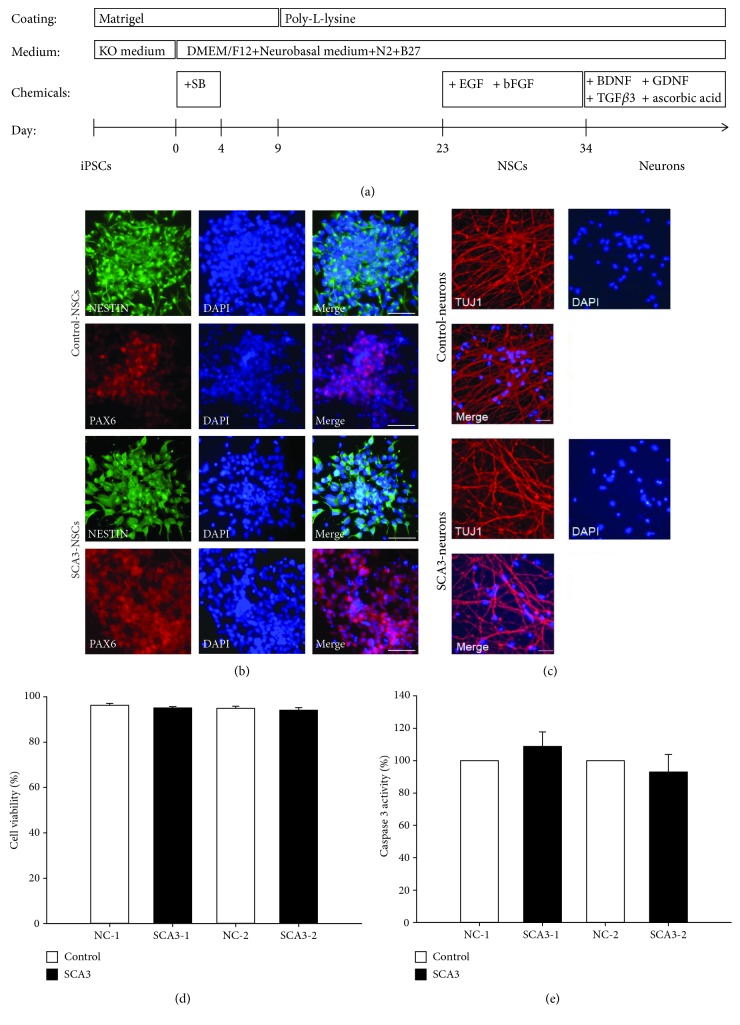
Differentiation of neural progenitors and neurons from control- and SCA3-iPSCs. (a) Flow chart of neuronal differentiation from iPSCs. KO: Knockout medium; SB: SB431542. (b) Neural stem cells (NSCs) positive to NESTIN (green) and PAX6 (red) were generated following three weeks of differentiation from iPSCs. (c) Matured neurons expressing TUJ1 (red) were obtained following another two weeks of differentiation from NSCs. Scale bar: 50 *μ*m. (d) Cell viability was determined by the trypan blue exclusion assay, and (e) caspase 3 activity was examined in the control- (NC-) and SCA3-iPSC-derived neurons. The caspase 3 activity in NC-iPSC-derived (NC-1 and NC-2) neurons was normalized as 100%. Each experiment for each sample was performed in triplicate.

Using a feeder-free monolayer differentiation protocol with defined chemicals (Figure 2(a)), these iPSCs became elongated and expressed NSC markers NESTIN and PAX6 four weeks after differentiation (Figure 2(b)). Mature neurons expressing TUJ1 were generated following another two weeks of differentiation (Figure 2(c)). The trypan blue exclusion test showed that neurons derived from SCA3- and NC-iPSCs demonstrated similar cell viability (ranging from 94 to 96%, *p* > 0.05, Figure 2(d)). Caspase 3 activities of neurons were also similar between SCA3 patients and NC subjects (NC set as 100%; NC-1 vs. SCA3-1: 100% vs. 109%, *p* > 0.05; NC-2 vs. SCA3-2: 100% vs. 93%, *p* > 0.05, Figure 2(e)).

### 3.2. Accumulation of PolyQ Aggregation in SCA3-iPSC-Derived Neurons

**Figure 3:**
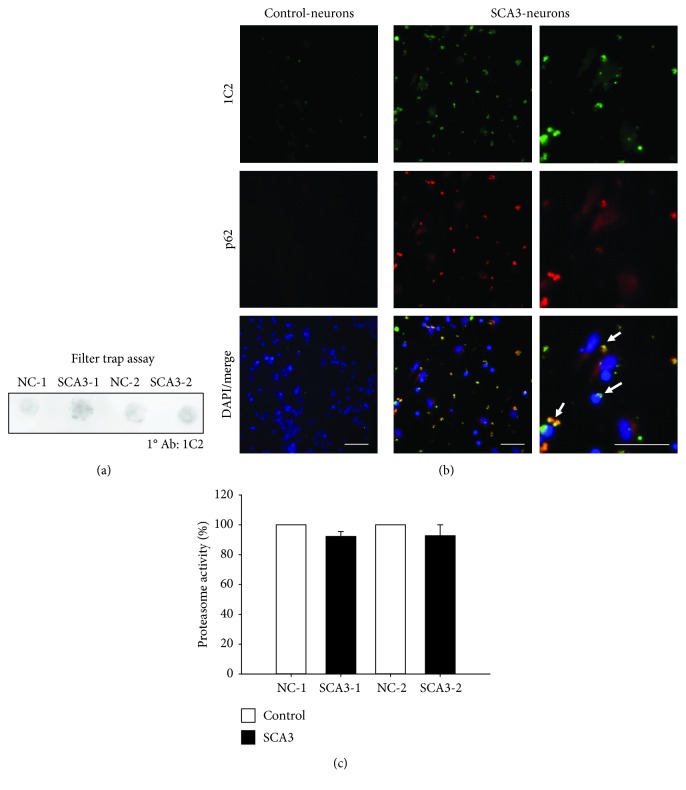
PolyQ aggregation and proteasome activity of SCA3-iPSC-derived neurons. (a) Filter trap assay was conducted with 1C2 antibody staining to detect the accumulation of polyQ aggregates. (b) SCA3-iPSC-derived neurons showed 1C2 staining-positive aggregates (green) and the aggregates colocalized with p62 (red) (white arrow). Scale bar: 50 *μ*m. (c) Proteasome activity assay was performed to analyze UPS function in iPSC-derived neurons. NC-iPSC-derived neurons (NC-1 and NC-2) were normalized as 100%. Each experiment for each sample was performed in triplicate.

To investigate whether SCA3-iPSC-derived neurons form polyQ aggregation, we performed a filter trap assay, which displayed greater intensity of aggregates positive to 1C2 staining, a marker for protein aggregation [23], in SCA3-iPSC-derived neurons compared to NC-iPSC-derived neurons (Figure 3(a)). Immunocytochemistry staining consistently showed a significant number of 1C2-positive aggregates in SCA3-iPSC-derived neurons, while these aggregates were not seen in NC-iPSC-derived neurons (Figure 3(b)). These 1C2-positive aggregates were located in the perinuclear region and also colocalized with P62, another marker for protein aggregation [24] (Figure 3(b)).

It has been shown that polyQ aggregation disturbed the UPS function [25]. The proteasome activity assay also showed that SCA3-iPSC-derived neurons had lower proteasome activity compared to NC-iPSC-derived neurons although the difference was not statistically significant (NC set as 100%; NC-1 vs. SCA3-1: 100% vs. 92%, *p* = 0.057; NC-2 vs. SCA3-2: 100% vs. 93%, *p* = 0.226, Figure 3(c)).

### 3.3. Proteasome Dysfunction and Increased Oxidative Stress in MG132-Stimulated SCA3-iPSC-Derived Neurons

**Figure 4:**
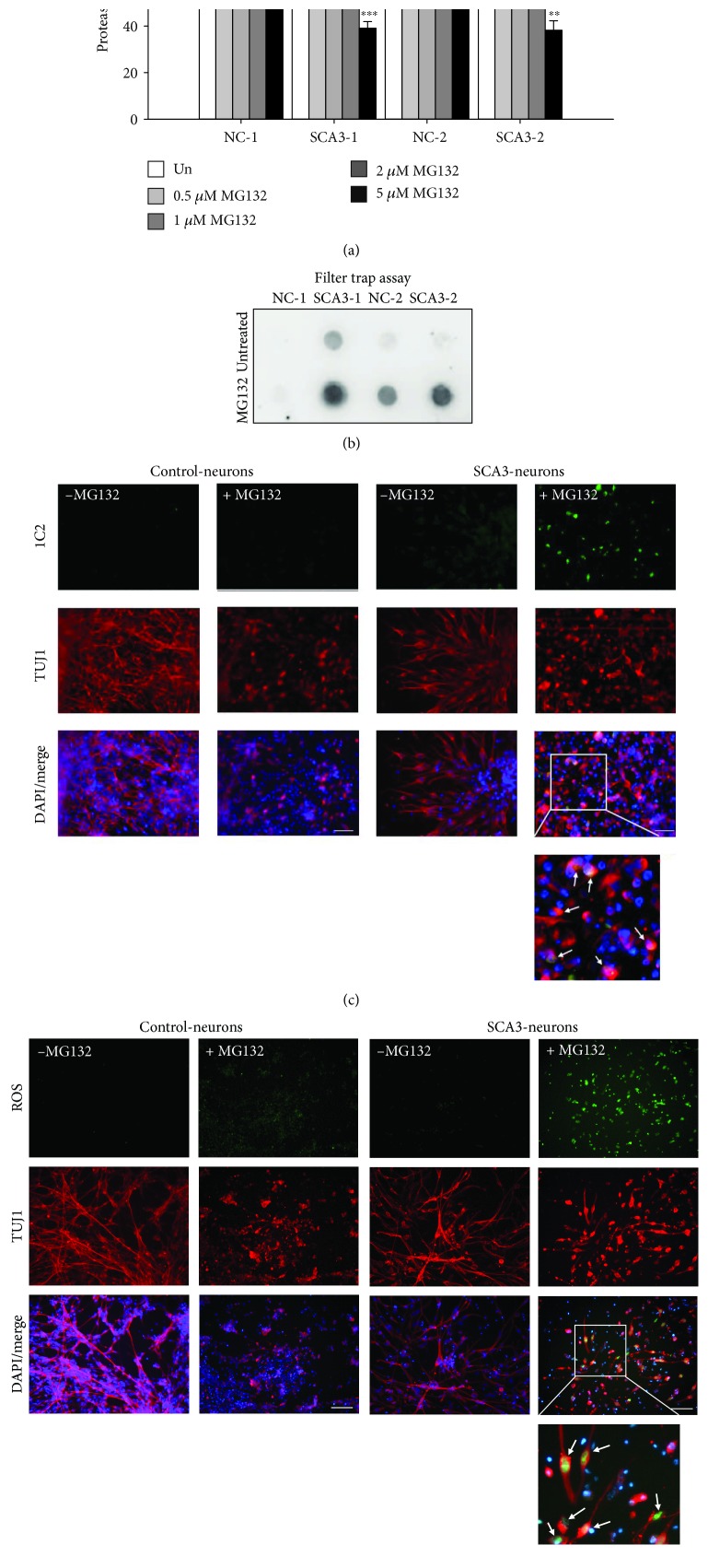
Impairment of the ubiquitin-proteasome system (UPS) in SCA3-iPSC-derived neurons treated with MG132. (a) Proteasome activity of iPSC-derived neurons treated with various concentrations of MG132 (0~5 *μ*M). Proteasome activity of untreated neurons was normalized as 100%. (b) 1C2-positive polyQ aggregates in 2 *μ*M MG132-treated iPSC-derived neurons were detected by the filter trap assay. (c) Representative images of iPSC-derived neurons stained with 1C2 (green) and anti-TUJ1 (red). The white arrows in the magnified picture indicate 1C2-positive aggregates colocalized with TUJ1. (d) Representative images of iPSC-derived neurons stained with a cellular ROS-detecting reagent (green). The white arrows in the magnified picture indicate ROS detected in TUJ1-positive neurons. Scale bar: 50 *μ*m. Each experiment for each sample was performed in triplicate. *p* values: MG132-treated vs. untreated, ^∗∗^*p* < 0.01 and ^∗∗∗^*p* < 0.001; SCA3-1 vs. NC-1 or SCA3-2 vs. NC-2, ^&^*p* < 0.05, ^&&^*p* < 0.01, and ^&&&^*p* < 0.001.

MG132 has been widely used as a potent proteasome inhibitor targeting 26S proteasome to block its activity [26]. To reveal the potential protein degradation impairment in SCA3, neurons derived from iPSCs were treated with MG132 (0~5 *μ*M) for 24 hours and then harvested for the proteasome activity assay. Treatment with MG132 significantly reduced proteasome activity in a dose-dependent manner in all iPSC-derived neurons (untreated set as 100%; 0~5 *μ*M MG132; NC-1: 100%~53%, *p* = 0.005~<0.001; SCA3-1: 100%~39%, *p* < 0.001; NC-2: 100%~50%, *p* = 0.002~<0.001; SCA3-2: 100%~38%, *p* = 0.005~<0.001, Figure 4(a)). Gender-matched comparison showed that the reduction of proteasome activity was greater in SCA3-iPSC-derived neurons compared to NC-iPSC-derived neurons (SCA3-1 vs. NC-1: 74% vs. 84% (0.5 *μ*M MG132), 64% vs. 72% (1 *μ*M MG132), 49% vs. 62% (2 *μ*M MG132), and 39% vs. 53% (5 *μ*M MG132), *p* = 0.034~<0.001; SCA3-2 vs. NC-2: 67% vs. 81% (0.5 *μ*M MG132), 59% vs. 71% (1 *μ*M MG132), 55% vs. 60% (2 *μ*M MG132), and 38% vs. 50% (5 *μ*M MG132), *p* = 0.049~0.014, Figure 4(a)). The filter trap assay also detected pronounced aggregates following 2 *μ*M MG132 treatment in SCA3-iPSC-derived neurons (Figure 4(b)). Treatment with 2 *μ*M MG132 specifically increased 1C2-positive aggregates in SCA3-iPSC-derived neurons, while the aggregation did not appear in NC-iPSC-derived neurons following MG132 treatment (Figure 4(c)). Treatment with 2 *μ*M MG132 further increased the amount of ROS in SCA3-iPSC-derived neurons compared to the NC-iPSC-derived neurons (Figure 4(d)).

### 3.4. SCA3-iPSC-Derived Neurons Demonstrating Increased Susceptibility to Proteasome Inhibitor

**Figure 5:**
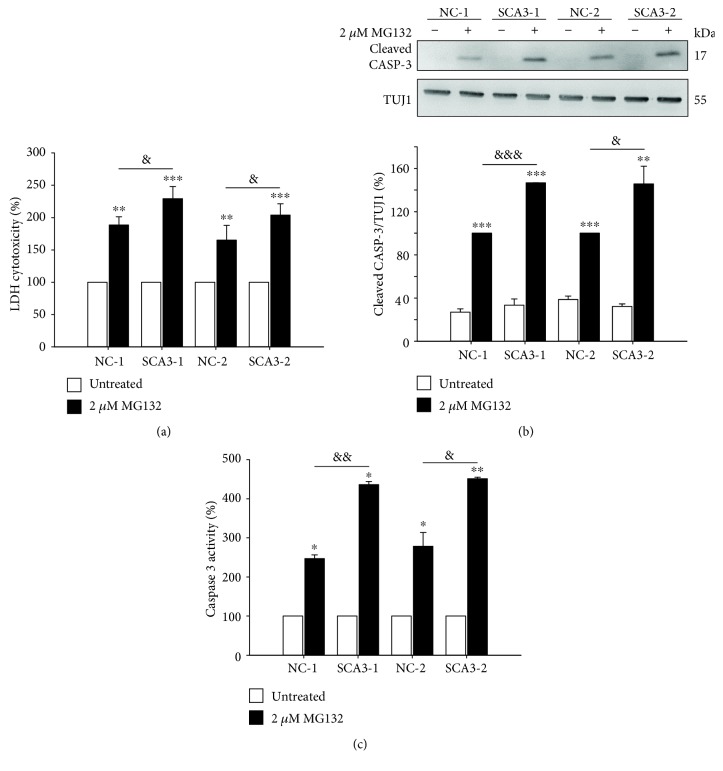
Elevated cytotoxicity and caspase 3 expression/activity in MG132-treated iPSC-derived neurons. (a) Lactic dehydrogenase (LDH) release in culture media was measured to evaluate cytotoxicity. LDH level of untreated neurons was normalized as 100%. (b) Western blot analysis of the cleaved caspase 3 level (normalized to TUJ1 as the internal control). Cleaved caspase 3 level of 2 *μ*M MG132-treated NC-iPSC-derived neurons (NC-1 and NC-2) was set as 100%. (c) Caspase 3 activity assay of NC- and SCA3-iPSC-derived neurons. Caspase 3 activity of untreated neurons was normalized as 100%. Each experiment for each sample was performed in triplicate. *p* values: MG132-treated vs. untreated, ^∗^*p* < 0.05, ^∗∗^*p* < 0.01, and ^∗∗∗^*p* < 0.001; SCA3-1 vs. NC-1 or SCA3-2 vs. NC-2, ^&^*p* < 0.05, ^&&^*p* < 0.01, and ^&&&^*p* < 0.001.

It has been reported that inhibition of proteasome function by MG132 induced apoptotic cell death in a Parkinson's disease (PD) iPSC model [20]. Using the LDH assay, we found that under 2 *μ*M MG132 treatment, SCA3-iPSC-derived neurons released a higher level of LDH compared to NC-iPSC-derived neurons (MG132-untreated neurons set as 100%, MG132-treated SCA3-1 vs. NC-1: 229% vs. 189%, *p* = 0.020; MG132-treated SCA3-2 vs. NC-2: 204% vs. 165%, *p* = 0.018, Figure 5(a)). Treatment with 2 *μ*M MG132 led to greater elevation of cleaved caspase 3 levels in both SCA3-iPSC-derived neurons compared to NC-iPSC-derived neurons (MG132-treated NC-1 and NC-2 set as 100%; MG132-treated SCA3-1 vs. NC-1: 147% vs. 100%, *p <* 0.001; MG132-treated SCA3-2 vs. NC-2: 146% vs. 100%, *p =* 0.040, Figure 5(b)). 2 *μ*M MG132 treatment also more greatly upregulated caspase 3 activities in SCA3-iPSC-derived neurons compared to NC-iPSC-derived neurons (MG132-untreated neurons set as 100%, MG132-treated SCA3-1 vs. NC-1: 436% vs. 247%, *p* = 0.002; MG132-treated SCA3-2 vs. NC-2: 451% vs. 278%, *p* = 0.012, Figure 5(c)). These results suggested that compared to the NC-iPSC-derived neurons, SCA3-iPSC-derived neurons were more susceptible to the MG132 treatment.

### 3.5. NH037 and Daidzein Enhancing Proteasome Function in iPSC-Derived Neurons

**Figure 6:**
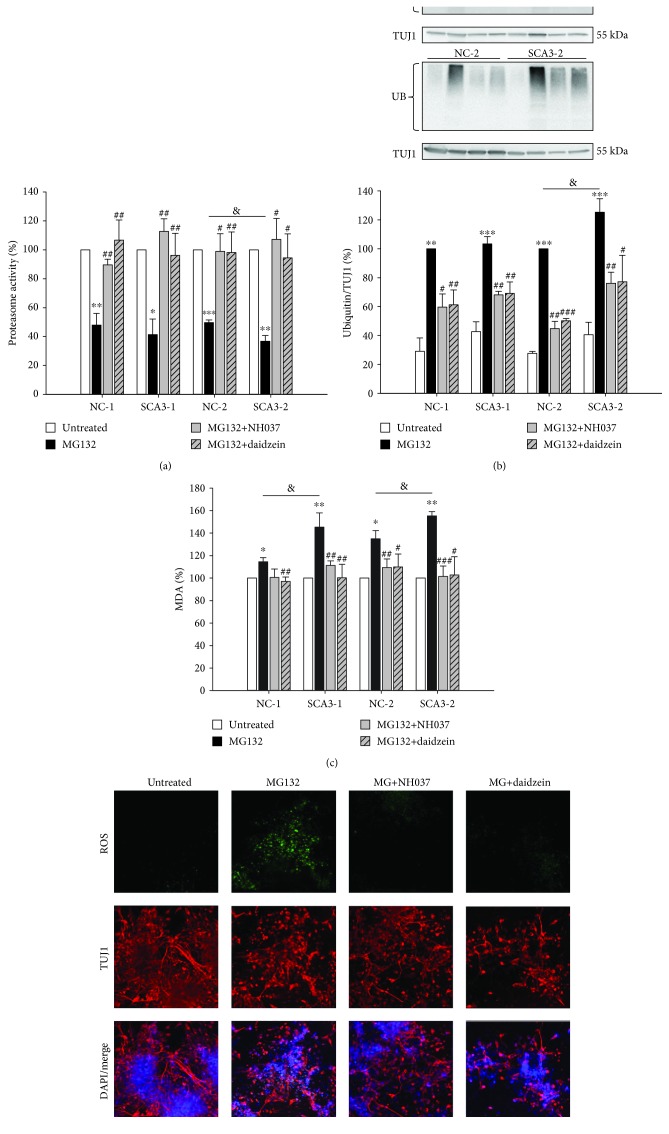
NH037 and daidzein promoted proteasome function and ameliorated oxidative stress in MG132-treated SCA3-iPSC-derived neurons. (a) Proteasome activity of iPSC-derived neurons treated with 2 *μ*M MG132 alone or MG132 together with 1 mg/ml NH037 or 50 *μ*M daidzein. The proteasome activity of untreated neurons was normalized as 100%. (b) Western blot analysis of ubiquitin. TUJ1 was used as the internal control. Ubiquitinated protein level of MG132-treated NC-iPSC-derived neurons (NC-1 and NC-2) was set as 100%. (c) Lipid peroxidation malondialdehyde (MDA) assay was conducted to evaluate oxidative stress. MDA level of untreated neurons was normalized as 100%. (d) Representative images of SCA3 neurons staining with TUJ1 (red) and cellular ROS detecting reagent (green) after treatment with 2 *μ*M MG132, 1 mg/ml NH037, or 50 *μ*M daidzein. Scale bar: 50 *μ*m. Each experiment for each sample was performed in triplicate. *p* values: MG132-treated vs. untreated, ^∗^*p* < 0.05, ^∗∗^*p* < 0.01, and ^∗∗∗^*p* < 0.001; SCA3-1 vs. NC-1 or SCA3-2 vs. NC-2, ^&^*p* < 0.05; MG132/NH037 vs. MG132 alone or MG132/daidzein vs. MG132 alone, ^#^*p* < 0.05, ^##^*p* < 0.01, and ^###^*p* < 0.001.

Previously, we found that Chinese herbal medicine NH037 and its constituent daidzein exerted neuroprotective effects by promoting proteasome function and reducing apoptotic protein expression in ATXN3/Q_75_-GFP expressing 293/SH-SY5Y cells. Previously, we have found that the IC_50_ value of NH037 and daidzein was >30 mg/ml and 100 *μ*M in ATXN3/Q_75_-GFP 293/SH-SY5Y cell models, suggesting low cytotoxicity of NH037 and daidzein [19]. Our previous study also showed that NH037 at 10 *μ*g/ml and daidzein at 0.1 *μ*M displayed neuroprotective effects on ATXN3/Q_75_-GFP SH-SY5Y cells [19]. According to these results and our observation that an effective concentration of a compound used to treat iPSC-derived neurons could be much higher than SH-SY5Y cells, we administered NH037 at 1 mg/ml and daidzein at 50 *μ*M to iPSC-derived neurons. We found that NH037 (1 mg/ml) slightly enhanced proteasome activity in NC- but not SCA3-iPSC-derived neurons (untreated set as 100%; untreated vs. NH037: 100% vs. 107%~118%, *p* = 0.014~0.207; untreated vs. daidzein: 100% vs. 109%~118%, *p* = 0.106~0.326, Supplementary [Supplementary-material supplementary-material-1]). Treatment with NH037 (1 mg/ml) or daidzein (50 *μ*M) rescued the downregulation of proteasome activities by 2 *μ*M MG132 in both NC- and SCA3-iPSC-derived neurons (untreated set as 100%; MG132 vs. MG132/NH037 or MG132/daidzein; NC-1: 48% vs. 90% or 107%, *p* = 0.005 or 0.007; SCA3-1: 41% vs. 113% or 96%, *p* = 0.001 or 0.003; NC-2: 50% vs. 99% or 98%, *p* = 0.018 or 0.006; SCA3-2: 37% vs. 107% or 94%, *p* = 0.010 or 0.022, Figure 6(a)). Treatment with 2 *μ*M MG132 produced a higher expression of smeared ubiquitinated proteins on both NC- and SCA3-iPSC-derived neurons (MG132-treated NC-1 and NC-2 set as 100%; MG132-treated vs. untreated; NC-1: 100% vs. 50%, *p* = 0.010; SCA3-1: 105% vs. 63%, *p* = 0.004; NC-2: 100% vs. 42%, *p* < 0.001; SCA3-2: 152% vs. 78%, *p* = 0.018, Figure 6(b)). The ubiquitination in 2 *μ*M MG132-treated neurons was also reduced by the treatment with 1 mg/ml NH037 or 50 *μ*M daidzein (MG132 vs. MG132/NH037 or MG132/daidzein; NC-1: 100% vs. 60% or 61%, *p* = 0.017 or 0.005; SCA3-1: 103% vs. 68% or 69%, *p* = 0.002 or 0.006; NC-2: 100% vs. 45% or 50%, *p* = 0.003 or <0.001; SCA3-2: 125% vs. 76% or 77%, *p* = 0.002 or 0.027, Figure 6(b)).

In NC-iPSC-derived neurons, treatment with daidzein (50 *μ*M) and not NH037 (1 mg/ml) affects levels of MDA, a lipid peroxidation marker [27], but either NH037 or daidzein has no effect on SCA3-iPSC-derived neurons (untreated set as 100%; untreated vs. NH037: 100% vs. 99%~104%, *p* = 0.072~0.971; untreated vs. daidzein: 100% vs. 97%~103%, *p* = 0.004~0.401, Supplementary [Supplementary-material supplementary-material-1]). Treatment with 2 *μ*M MG132 further generated higher levels of MDA compared to untreated cells (untreated set as 100%; MG132-treated: NC-1: 115%, *p* = 0.021; SCA3-1: 145%, *p* = 0.006; NC-2: 135%, *p* = 0.014; SCA3-2: 155%, *p* = 0.002, Figure 6(c)). 1 mg/ml NH037 or 50 *μ*M daidzein significantly normalized the MDA level under 2 *μ*M MG132 treatment (MG132 vs. MG132/NH037 or MG132/daidzein; NC-1: 115% vs. 101% or 97%, *p* = 0.064 or 0.005; SCA3-1: 145% vs. 111% or 100%, *p* = 0.009 or 0.006; NC-2: 135% vs. 109% or 110%, *p* = 0.007 or 0.017; SCA3-2: 155% vs. 101% or 103%, *p* < 0.001 or =0.025). Importantly, treatment with 1 mg/ml NH037 or 50 *μ*M daidzein significantly reduced ROS levels in SCA3-iPSC-derived neurons (Figure 6(d)).

### 3.6. NH037 and Daidzein Reducing Cytotoxicity in iPSC-Derived Neurons

**Figure 7:**
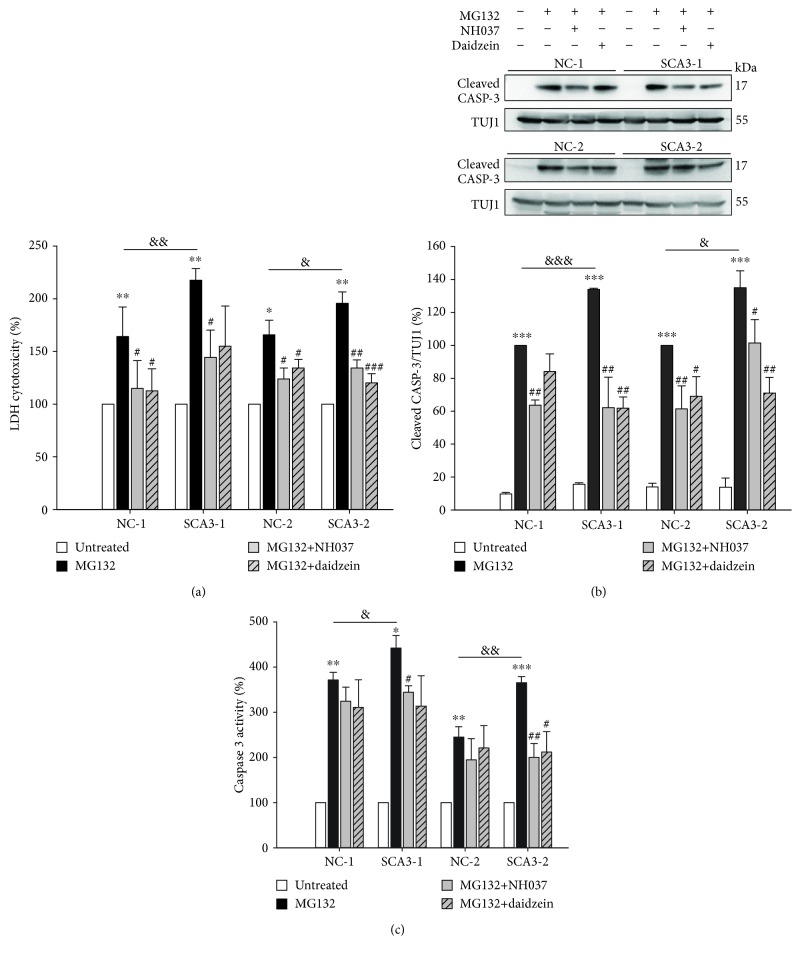
NH037 and daidzein reduced cytotoxicity and caspase 3 expression/activity in MG132-treated SCA3-iPSC-derived neurons. (a) LDH assay in iPSC-derived neurons treated with 2 *μ*M MG132 alone or MG132 and 1 mg/ml NH037/50 *μ*M daidzein (untreated neurons were normalized as 100%). (b) Western blot analysis of cleaved caspase 3 (normalized to TUJ1 as the internal control) in iPSC-derived neurons treated with 2 *μ*M MG132 alone or MG132 and 1 mg/ml NH037/50 *μ*M daidzein. The levels of cleaved caspase 3 from MG132-treated NC-iPSC-derived neurons (NC-1 and NC-2) were set as 100%. (c) Caspase 3 activity in iPSC-derived neurons treated with 2 *μ*M MG132 alone or MG132 and 1 mg/ml NH037/50 *μ*M daidzein. Untreated neurons were normalized as 100%. Each experiment for each sample was performed in triplicate. *p* values: MG132-treated vs. untreated, ^∗^*p* < 0.05, ^∗∗^*p* < 0.01, and ^∗∗∗^*p* < 0.001; SCA3-1 vs. NC-1 or SCA3-2 vs. NC-2, ^&^*p* < 0.05, ^&&^*p* < 0.01, and ^&&&^*p* < 0.001; MG132/NH037 vs. MG132 alone or MG132/daidzein vs. MG132 alone, ^#^*p* < 0.05, ^##^*p* < 0.01.

Treatment with NH037 (1 mg/ml) and not daidzein (50 *μ*M) slightly decreased release of LDH in SCA3-iPSC-derived neurons, whereas either NH037 or daidzein has no effect on NC-iPSC-derived neurons (untreated set as 100%; untreated vs. NH037: 100% vs. 83%~90%, *p* = 0.004~0.381; untreated vs. daidzein: 100% vs. 97%~104%, *p* = 0.612~0.949, Supplementary [Supplementary-material supplementary-material-1]). Treatment with 1 mg/ml NH037 or 50 *μ*M daidzein significantly ameliorated the 2 *μ*M MG132-induced abnormal release of LDH in both NC- and SCA3-iPSC-derived neurons (untreated set as 100%; MG132 vs. MG132/NH037 or MG132/daidzein; NC-1: 164% vs. 115% or 113%, *p* = 0.026 or 0.024; SCA3-1: 218% vs. 144% or 155%, *p* = 0.025 or 0.094; NC-2: 166% vs. 124% or 134%, *p* = 0.016 or 0.038; SCA3-2: 196% vs. 134% or 120%, *p* = 0.002 or <0.001, Figure 7(a)).

2 *μ*M MG132-induced upregulation of cleaved caspase 3 was also reduced by treatment with 1 mg/ml NH037 or 50 *μ*M daidzein (MG132-treated NC-1 and NC-2 set as 100%; MG132 vs. MG132/NH037 or MG132/daidzein; NC-1: 100% vs. 64% or 84%, *p* = 0.003 or 0.088; SCA3-1: 134% vs. 62% or 62%, *p* = 0.001 or 0.003; NC-2: 100% vs. 61% or 69%, *p* = 0.003 or 0.014; SCA3-2: 135% vs. 101% or 71%, *p* = 0.015 or 0.001, Figure 7(b)). Treatment with 1 mg/ml NH037 and not 50 *μ*M daidzein slightly affects caspase 3 activity in SCA3-iPSC-derived neurons, whereas either NH037 or daidzein has no effect on NC-iPSC-derived neurons (untreated set as 100%; untreated vs. NH037: 100% vs. 87%~106%, *p* = 0.003~0.684; untreated vs. daidzein: 100% vs. 101%~113%, *p* = 0.189~0.978, Supplementary [Supplementary-material supplementary-material-1]). Treatment of 1 mg/ml NH037 significantly decreased MG132-induced upregulation of caspase 3 activity specifically in SCA3-iPSC-derived neurons (untreated set as 100%; MG132 vs. MG132/NH037; SCA3-1: 442% vs. 344%, *p* = 0.016; SCA3-2: 365% vs. 200%, *p* = 0.004), while 50 *μ*M daidzein reduced caspase 3 activity only in SCA3-2-derived neurons (untreated set as 100%, MG132 vs. MG132/daidzein; 365% vs. 212%, *p* = 0.020) (Figure 7(c)).

## 4. Discussion

**Figure 8:**
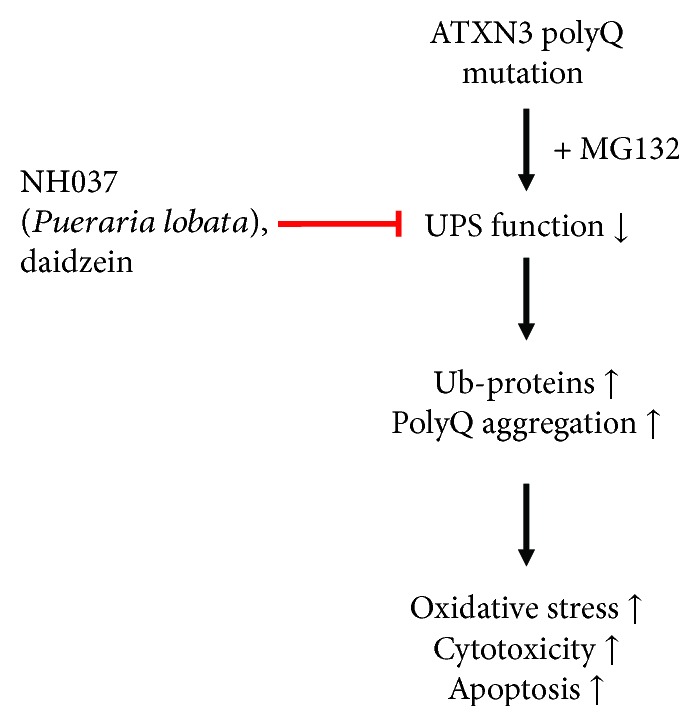
Hypothesis of NH037 and daidzein targeting UPS function in SCA3-iPSC-derived neurons. In SCA3-iPSC-derived neurons, UPS function is impaired under environmental stress (such as MG132 exposure), and ubiquitinated proteins and polyQ-expanded ATXN3 protein are accumulated in cells. The resulting abnormal aggregation increased the production of oxidative stress and cytotoxicity. NH037 and daidzein promote protein degradation to reduce oxidative stress and cytotoxicity by improving proteasome activity in SCA3-iPSC-derived neurons.

In this study, we successfully established iPSC lines and iPSC-derived neurons from patients with SCA3 (Figures 1 and 2). SCA3-iPSC-derived neurons showed abnormal intracellular aggregates, decreased proteasome function, pronounced ROS, and prominent cytotoxicity under proteasome inhibition by MG132 (Figures 3–5). NH037 or daidzein provided neuroprotection against MG132 cytotoxicity through enhancing UPS and suppressing the production of ROS on SCA3-iPSC-derived neurons (Figures 6–8). These findings indicate an important role of gene-environmental interaction in SCA3-mediated neurodegeneration, as well as the potential of proteasome enhancer in treating SCA3.

The rapid development in the iPSC field and the potential of iPSC to differentiate into different cell types not only offer great opportunities to study the pathogenesis of neurodegenerative diseases including SCA3 but also provide a platform to identify new therapeutic strategies [28–31]. In the past decade, neuronal cells derived from patient-specific iPSCs have been generated to model neurodegenerative diseases such as PD [32, 33], Alzheimer's disease (AD) [34, 35], and HD [36, 37], and relatively handful iPSC studies focused on cerebellar ataxias including Friedreich's ataxia (FRDA) [38–43] and SCAs: SCA2 [44], SCA3 [45–48], SCA6 [49], SCA7 [50], SCA14 [51], and SCA36 [52]. Koch et al. first reported that L-glutamate-induced excitation of SCA3-iPSC-derived neurons initiated calpain-dependent proteolysis of ATXN3 followed by the formation of SDS-insoluble aggregates [46]. However, glutamate-induced ATXN3 aggregation was not observed in SCA3-iPSC-derived neurons generated by other groups using different differentiation protocols [45, 48]. A high concentration of db-cAMP in their neuronal differentiation medium may reduce the formation of aggregates by increasing proteasome activity [45, 53]. We performed neural differentiation of iPSCs without db-cAMP treatment, and 1C2-positive aggregates were detected in SCA3-iPSC-derived neurons (Figure 3(b)). Consistent with pervious findings [46, 48], SCA3- and control-iPSC-derived neurons displayed similar cell viability without MG132 treatment (Figure 2(d)). However, treating neurons with MG132 resulted in lower cell viability, higher caspase 3 activity, impaired UPS function, and pronounced ROS in SCA3-iPSC-derived neurons (Figures 4 and 5), suggesting the important role of environmental stress in ATXN3-related neurodegeneration. The gene-environmental interactions have been recapitulated by different iPSC models for neurodegenerative diseases. Treatment with MG132 has been reported to induce cell death in iPSC-derived neurons carrying *PARKIN* mutation for PD [20]. Stressors such as cytokine treatment, oxidative stress, proteasome inhibition, or growth factor withdrawal are mandatory to generate phenotypes such as cell death, caspase 3 activation, or aggregate formation in HD-iPSC models [54–59]. Nutrient-depletion stress induced degeneration of dendrites in the iPSC-derived neurons from SCA6 patients [49]. These studies including our findings suggest the pivotal roles of environmental triggers in neurodegeneration. In this study, MG132 was used to trigger more aggregation, oxidative stress, and cytotoxicity in SCA3-iPSC-derived neurons, but proteasome inhibition could activate autophagy as a compensatory reaction during the cell recovery process [60, 61]. Since our study did not examine the markers of autophagy, whether MG132-induced activation of autophagy occurs in the SCA3-iPSC-derived neurons remains to be clarified. It is worthy to mention that MG132 treatment on SCA3-iPSC-derived neurons still exacerbates aggregation, oxidative stress, and apoptosis, suggesting that even if there is an autophagy induction, it is not able to overcome the cytotoxicity induced by proteasome inhibition. In addition, although our previous study has shown no effect of NH037 and daidzein on autophagy in 293 cells [19], the autophagy-enhancing activity by NH037 and daidzein in our SCA3-iPSC model could not be excluded. Therefore, it is important to investigate if NH037 and/or daidzein also exert neuroprotection via acting on autophagy in SCA3-iPSC-derived neurons in the future.

While iPSC models provided insights into the pathogenesis of diseases, several studies have examined therapeutic applications in SCA3-iPSC models. Autophagy inducer, rapamycin, promoted autophagy and degraded expanded polyQ ATXN3 in neurons differentiated from SCA3-iPSCs [47]. Formation of SDS-insoluble aggregates in SCA3-derived neurons can be suppressed by calpain inhibition [46]. However, none of these treatments demonstrated neuroprotective effects on SCA3-iPSC-derived neurons. Our study, for the first time, demonstrates neuroprotective potentials of NH037 or daidzein by enhancing UPS to suppress oxidative stress, cytotoxicity, and apoptosis (Figures 6 and 7).

NH037 is an herbal extract prepared from *P. lobata* that is used traditionally to treat diarrhea, muscle stiffness, thirst, and diabetes in East Asia, and *P. lobata* has also been studied in several diseases including neurodegenerative diseases [62]. The main active constituents of *P. lobata* are isoflavonoids, which exhibit a wide range of biological activities including anti-inflammatory, antithrombotic, antihypertensive, antiarrhythmic, spasmolytic, and cancer chemopreventive properties [63]. Isoflavonoids also have demonstrated prominent neuroprotective effects against cerebrovascular disorders, hypertension, or PD in rats or cell models [64–66]. One of its most abundant isoflavonoids, daidzein [67, 68], exerts neuroprotective effect to enhance neuronal survival and has been implicated to be a potential drug for neurodegenerative diseases [69–71]. Treatment with daidzein in PC12 cells reduced A*β* aggregates and the neuronal cytotoxicity of A*β* [69]. In primary rat dorsal root ganglion (DRG) neuronal cultures, daidzein enhanced neuritogenesis depending on Src kinase, PKC*δ*, and ERK signaling pathway [71]. Daidzein also improved neuronal cell viability and proliferation *in vitro* through activating the BDNF-TRK pathway [70]. In the SCA3-iPSC model, our study showed the neuroprotective effect of daidzein mediated by enhancing UPS, further indicating its pleiotropic effects on protecting neurons against different neurodegenerative pathogenesis.

## 5. Conclusions

In conclusion, this is the first study demonstrating UPS impairment in SCA3-iPSC-derived neurons and the neuroprotective potentials of NH037 and its constituent daidzein in SCA3. Recently, CRISPR/Cas9 genomic editing has been used to successfully eliminate the expanded polyQ in SCA3 patient-derived iPSCs [48]. It would be important to test the effects of NH037 and daidzein on SCA3-iPSC-derived and CRISPR/Cas9-corrected isogenic SCA3-iPSC-derived neurons to eliminate the variations caused by different genetic backgrounds. The acquisition of Purkinje cells from SCA3-iPSCs will further elucidate how the mutant ATXN3 selectively causes cerebellar degeneration [72]. Nevertheless, our findings shed lights on identification of new therapeutic compounds targeting UPS in SCA3. Further validation using various animal models will be warranted before translating our findings to clinical studies.

## Data Availability

The data used to support the findings of this study are available from the corresponding author upon request.

## Abbreviations

AD: Alzheimer's disease
CHM: Chinese herbal medicine
DMEM: Dulbecco's modified Eagle medium
DRG: Dorsal root ganglion
FRDA: Friedreich's ataxia
HD: Huntington's disease
iPSC: Induced pluripotent stem cell
KSR: Knockout Serum Replacement
LDH: Lactate dehydrogenase
MDA: Malondialdehyde
MEFs: Mouse embryonic fibroblasts
MJD: Machado-Joseph disease
NSC: Neural stem cell
PD: Parkinson's disease
PolyQ: Polyglutamine
ROS: Radical oxygen species
SCA3: Spinocerebellar ataxia type 3
UPS: Ubiquitin-proteasome system.

## Supplementary Materials

Supplementary Materials Supplementary Figure 1: effects of NH037 and daidzein on proteasome function and oxidative stress in NC- or SCA3-iPSC-derived neurons. (a) Proteasome activity of iPSC-derived neurons treated with 1 mg/ml NH037 or 50 *μ*M daidzein. The proteasome activity of untreated neurons was normalized as 100%. (b) Lipid peroxidation MDA assay was conducted to evaluate oxidative stress. MDA level of untreated neurons was normalized as 100%. Each experiment for each sample was performed in triplicate. *p* values: NH037- or daidzein-treated vs. untreated, ^∗^*p* < 0.05 and ^∗∗^*p* < 0.01. Supplementary Figure 2: the effects of NH037 and daidzein on cytotoxicity and caspase 3 activity in NC- or SCA3-iPSC-derived neurons. (a) LDH assay in iPSC-derived neurons treated with 1 mg/ml NH037 or 50 *μ*M daidzein (untreated neurons was normalized as 100%). (b) Caspase 3 activity in iPSC-derived neurons treated with 1 mg/ml NH037 or 50 *μ*M daidzein. Untreated neurons were normalized as 100%. Each experiment for each sample was performed in triplicate. *p* values: NH037- or daidzein-treated vs. untreated, ^∗∗^*p* < 0.01.
