# Supplementary material for: Pueraria lobata and Daidzein Reduce Cytotoxicity by Enhancing Ubiquitin-Proteasome System Function in SCA3-iPSC-Derived Neurons
Source: Oxid Med Cell Longev. 2019 Oct 7;2019:8130481. doi: 10.1155/2019/8130481 (PMC6800904; doi:10.1155/2019/8130481)
Supplement: Supplementary Materials — Supplementary Figure 1: effects of NH037 and daidzein on proteasome function and oxidative stress in NC- or SCA3-iPSC-derived neurons. (a) Proteasome activity of iPSC-derived neurons treated with 1 mg/ml NH037 or 50 μM daidzein. The proteasome activity of untreated neurons was normalized as 100%. (b) Lipid peroxidation MDA assay was conducted to evaluate oxidative stress. MDA level of untreated neurons was normalized as 100%. Each experiment for each sample was performed in triplicate. p values: NH037- or daidzein-treated vs. untreated, ∗p < 0.05 and ∗∗p < 0.01. Supplementary Figure 2: the effects of NH037 and daidzein on cytotoxicity and caspase 3 activity in NC- or SCA3-iPSC-derived neurons. (a) LDH assay in iPSC-derived neurons treated with 1 mg/ml NH037 or 50 μM daidzein (untreated neurons was normalized as 100%). (b) Caspase 3 activity in iPSC-derived neurons treated with 1 mg/ml NH037 or 50 μM daidzein. Untreated neurons were normalized as 100%. Each experiment for each sample was performed in triplicate. p values: NH037- or daidzein-treated vs. untreated, ∗∗p < 0.01. [file 8130481.f1.pdf]

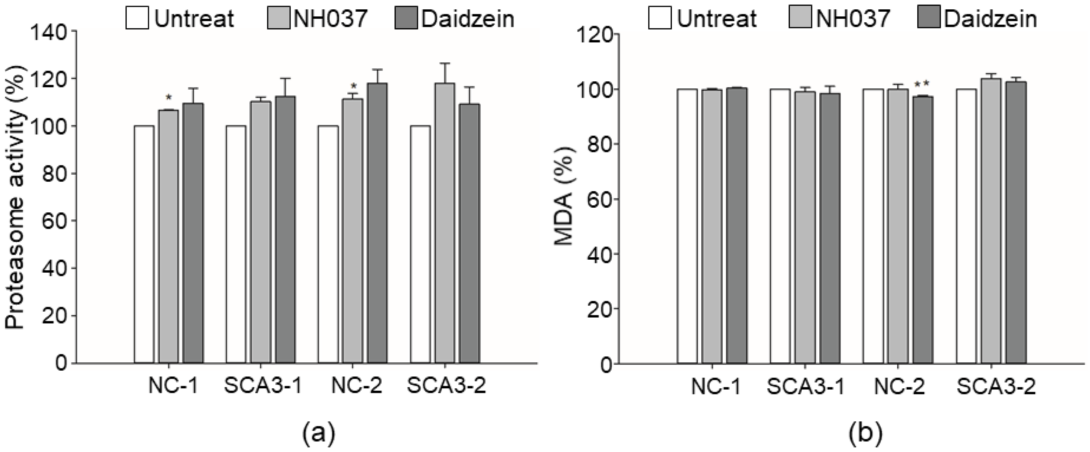

SUPPLEMENTARY FIGURE 1: Effects of NH037 and daidzein on proteasome function and oxidative stress in NC-, or SCA3-iPSC-derived neurons. (a) Proteasome activity of iPSC-derived neurons treated with 1 mg/ml NH037 or 50 µM daidzein. The proteasome activity of untreated neurons was normalized as 100%. (b) Lipid peroxidation MDA assay was conducted to evaluate oxidative stress. MDA level of untreated neurons was normalized as 100%. Each experiment for each sample was performed in triplicate. *P* values, NH037 or daidzein treated vs. untreated, $^{*}p < 0.05$ and $^{**}p < 0.01$.

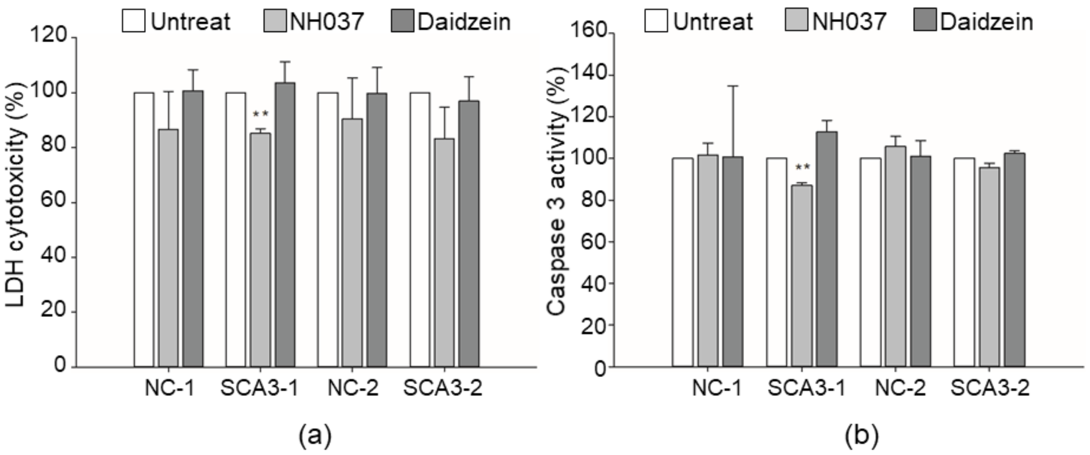

SUPPLEMENTARY FIGURE 2: The effects of NH037 and daidzein on cytotoxicity and caspase 3 activity in NC-, or SCA3-iPSC-derived neurons. (a) LDH assay in iPSC-derived neurons treated with 1 mg/ml NH037 or 50 μM daidzein (untreated neurons was normalized as 100%). (b) Caspase 3 activity in iPSC-derived neurons treated with 1 mg/ml NH037 or 50 μM daidzein. Untreated neurons were normalized as 100%. Each experiment for each sample was performed in triplicate. *P* values, NH037 or daidzein treated vs. untreated, $^{**}p < 0.01$.
